# Multifocal Electroretinogram Can Detect the Abnormal Retinal Change in Early Stage of type2 DM Patients without Apparent Diabetic Retinopathy

**DOI:** 10.1155/2021/6644691

**Published:** 2021-02-23

**Authors:** Jiang Huang, Yi Li, Yao Chen, Yuhong You, Tongtong Niu, Weijie Zou, Weifeng Luo

**Affiliations:** ^1^Department of Ophthalmology, Second Affiliated Hospital of Soochow University, China; ^2^Department of Ophthalmology, Huashan Hospital North, Fudan University, China; ^3^Department of Neurology, Second Affiliated Hospital of Soochow University, China

## Abstract

**Purpose:**

To study retinal function defects in type 2 diabetic patients without clinically apparent retinopathy using a multifocal electroretinogram (mf-ERG).

**Methods:**

Seventy-six eyes of thirty-eight type 2 diabetes mellitus(DM) patients without clinically apparent retinopathy and sixty-four normal eyes of thirty-two healthy control (HC) participants were examined using mf-ERG.

**Results:**

Patients with type 2 DM without apparent diabetic retinopathy demonstrated an obvious implicit time delay of P1 in ring 1, ring 3, and ring 5 compared with healthy controls (*t* = 5.184, *p* ≤ 0.001; *t* = 8.077, *p* ≤ 0.001; *t* = 2.000, *p* = 0.047, respectively). The implicit time (IT) in ring 4 of N1wave was significantly delayed in the DM group (*t* = 2.327, *p* = 0.021). Compared with the HC group, the implicit time of the P1 and N1 waves in the temporal retina zone was significantly prolonged (*t* = 3.66, *p* ≤ 0.001; *t* = 2.187, *p* = 0.03, respectively). And the amplitude of P1 in the temporal retina decreased in the DM group, which had a significantly statistical difference with the healthy controls (*t* = −6.963, *p* ≤ 0.001). However, there were no differences in either the amplitude of the response or the implicit time of the nasal retina zone between DM and HC. The AUC of multiparameters of mf-ERG was higher in the diagnosis of DR patients.

**Conclusions:**

Patients with type 2 DM without clinically apparent retinopathy had a delayed implicit time of P1 wave in temporal regions of the postpole of the retina compared with HC subjects. It demonstrates that mf-ERG can detect the abnormal retinal change in the early stage of type2 DM patients without apparent diabetic retinopathy. Multiparameters of mf-ERG can improve the diagnostic efficacy of DR, and it may be a potential clinical biomarker for early diagnosis of DR.

## 1. Introduction

Diabetic retinopathy (DR) is one of the irreversible blindness in working-age people in mainland China [1, 2]. Laser photocoagulation, intraocular drug injections (including anti-VEGF), and vitrectomy are the major therapies used to treat DR. All these therapies slow the progression of retinopathy; however, they are unlikely to reverse the loss of vision, and the effective methods for saving vision in the late stages of diabetic retinopathy are still lacking. Moreover, during the stage of proliferative diabetic retinopathy, patients often experience great difficulty in performing daily life activities, and vision-related quality of life shows a particularly dramatic decline.

Previous studies [3–6] have shown that the mf-ERG is a noninvasive and specific method for detecting the changes of the retinal function in diabetic retinopathy. The delayed implicit time of mf-ERG is locally predictive of nonproliferative retinopathy [7–9]. Additionally, a large amount of research has suggested that neural damage occurs in the retina before the retinal vascular changes become apparent [10–12].

The detection of changes in the retinal function before the occurrence of diabetic damage to the retina would provide significant clinical evidence for early intervention. The purpose of this study was to identify abnormalities in the retinal function in Chinese type 2 diabetic patients without clinically apparent retinopathy by mf-ERG examination.

## 2. Methods

### 2.1. Study Subjects

The study design complied with the principles of the Declaration of Helsinki and all procedures were approved by the Committee on Human Studies of The Second Affiliated Hospital of Soochow University. Written consent was obtained from the participants regarding the use of their clinical records. Seventy-six eyes of thirty-eight type 2 diabetes mellitus (DM) patients without clinically apparent retinopathy (mean age, 64.08 ± 8.53 years) based on several ocular examinations, including slit-lamp, ophthalmoscopy, noncontact intraocular pressure and fundus photography, and sixty-four normal eyes from thirty-two healthy control (HC) participates (mean age, 65.19 ± 5.46 years), were randomly examined using multifocal electroretinogram. The duration of diabetes ranged from 5 to 10 years (mean = 7.13 ± 1.63 years). All eyes had a visual acuity above 16/20 without apparent microaneurysm or exudation in the retina. Patients with glaucoma, hypermyopia, macular disease, and other fundus diseases were excluded from the study.

A review of medical records and an ocular examination revealed that all normal subjects were free of ocular and systemic disease and had a corrected visual acuity of 16/20 or better with a refractive error range from +1.00D to -2.50D. The potential risks and purposes of the study were explained to the subjects, and informed consent was obtained from all subjects before testing. The procedures followed the tenets of the Second Affiliated Hospital of Soochow University Committee for the protection of human subjects.

### 2.2. mf-ERG Recording

**Figure 1:**
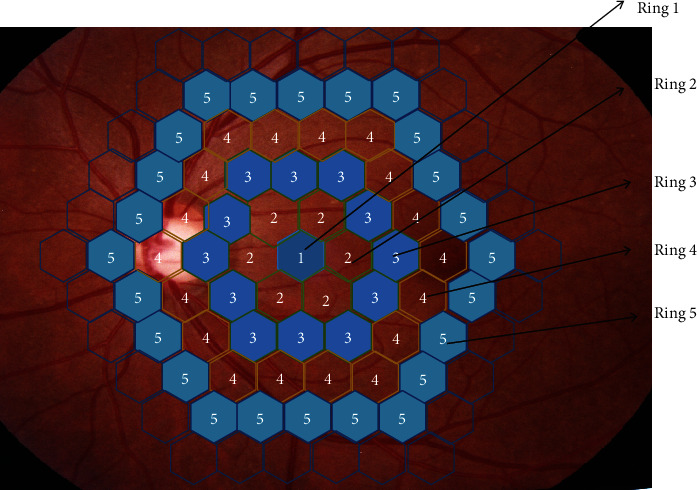
shows a fundus photo of the retina with the five rings regions where the mf-ERG can detect the retinal function in the relevant area by extraction of signal in posterior retina.

mf-ERG was performed followed by the International Society for Clinical Electrophysiology of Vision guidelines with the test system (VETS V8.1; GOTEC, Chongqing). Pupils of participants were dilated (≥7 mm) using 1.0% tropicamide and 2.5% phenylephrine. After topical corneal anesthesia (0.5% proparacaine), a monopolar Jet contact len electrode was used. Patients were positioned in front of a 19-inch CRT monitor with a distance of 33 cm. A scaled 103-hexagon stimulus pattern with a frame rate of 75 Hz was shown on the CRT monitor. With an m-sequence in every 8-minute recording cycle, the hexagons were regulated between white (200 cd/m^2^) and black (<2 cd/m^2^).The stimulus area was focused on the fovea of the posterior retina (Figure 1), and the data recording was collected in approximately 25 seconds. A Burian-Allen contact len electrodes were placed on the anesthetized (0.4% hydrochloride Oxybuprocaine) cornea surface of both eyes. The ground contact electrode was placed on the right earlobe, and the electrode impedance was kept below 5 k*Ω*. Fixation was controlled using an “x” target in the center of the stimulus [13]. Contaminated segments were discarded and reevaluated. The amplitude density (AD) and implicit time (IT) were used to analyze general information.

### 2.3. Statistical Analysis

The Mann–Whitney *U* test was used to identify significant differences between the diabetes mellitus (DM) group and the healthy control (HC) group for all measurements (AD and IT). The results are presented as mean ± SD. Student's two-tailed *t*-test was used to compare age. The statistical analyses were performed with the SPSS21.0 (SPSS Inc., Chicago, USA). All statistical tests were two-tailed, and a *p* < 0.05 was considered statistically significant. The Pearson chi-square test was used for comparison of sex.

## 3. Results

### 3.1. General Demographic Analysis

The demographic data of all participants are shown in Table 1.There were no statistically significant differences for age, sex, BCVA, and intraocular pressure (IOP) between the HC and DM group. However, compared with the HC group, the glycosylated hemoglobin (HbA1c) in the DM group was significantly higher in the DM group (*p* < 0.01).

### 3.2. Multifocal Electroretinography in Rings

The amplitude density (AD) of P1 wave did not differ significantly between the healthy controls and the diabetic patients for all rings (Table 2). And there was no statistically significant difference in the amplitude of N1 wave. Compared with the HC group, the implicit time (IT) in ring 1, ring 3, and ring 5 of the P1 wave was significantly prolonged in type 2 diabetic patients (*t* = 5.184, *p* ≤ 0.001; *t* = 8.077, *p* ≤ 0.001; *t* = 2.000, *p* = 0.047, respectively). The implicit time (IT) in ring4 of N1wave was significantly delayed in the DM group (*t* = 2.327, *p* = 0.021).

### 3.3. Multifocal Electroretinography in the Temporal and Nasal Retina Zone

**Figure 2:**
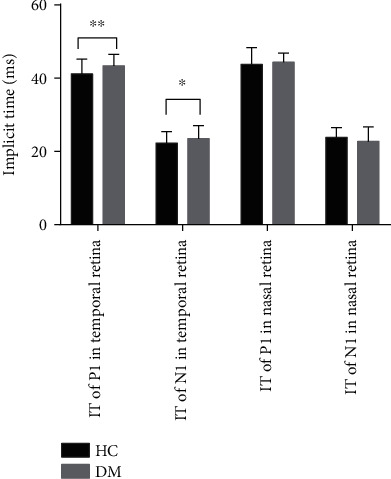
The implicit time of P1 and N1 waves in temporal and nasal retina between HC and DM patients.

**Figure 3:**
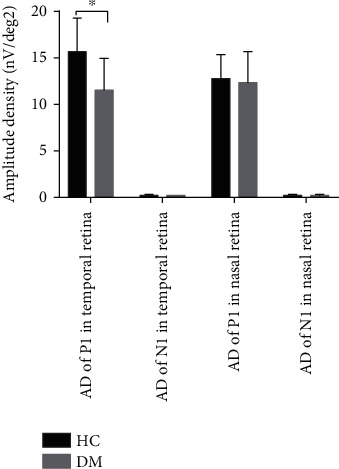
The amplitude of P1 and N1 waves in temporal and nasal retina between HC and DM patients, ^∗^*p* < 0.05.

Compared with the control group, the implicit time of the P1 and N1 waves, which reflected the function of the temporal retina zone, was significantly prolonged (*t* = 3.66, *p* ≤ 0.001; *t* = 2.187, *p* = 0.03, respectively) (Figure 2). And the amplitude of P1 in temporal retina decreased in the DM group, which had a significantly statistical difference with the healthy control (*t* = −6.963, *p* ≤ 0.001) (Figure 3). However, there were no differences in either the amplitude of the response or the implicit time of the nasal retina zone between the patient group and the control group (Figures 2 and 3).

### 3.4. The ROC Curve Analyses of mf-ERG Examination Results to Test the Predictive Ability of Diabetic Retinopathy

**Figure 4:**
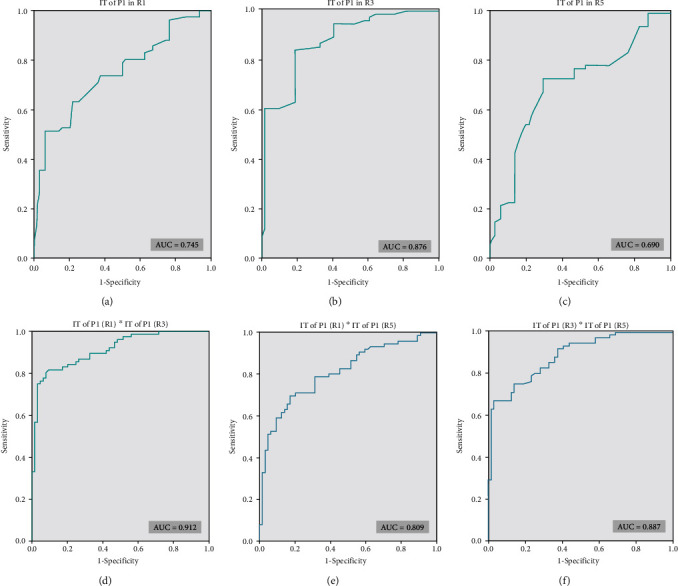
(a)–(c) shows ROC curves of the single parameter of mf-ERG for diagnosing diabetic retinopathy. (d)–(f) shows ROC curves of multiparameters of mf-ERG for diagnosing diabetic retinopathy. IT: implicit time; R1: ring 1; R3: ring 3; R5: ring 5.

The ROC curves of a single indicator of mf-ERG were represented in Figures 4(a)–4(c). The ROC curves of P1 in ring 1,ring 3, and ring 5 to detect DM were 0.745, 0.876, and 0.690, respectively.

The ROC curves of combinative indicators were plotted in Figures 4(d)–4(f). The AUC of a combination of the IT of P1 in ring 1 and that in ring 3 to detect DM diagnosis DM was 0.912. A combination of the IT of P1 in ring 1 and that in ring 5 revealed an AUC of 0.809. The AUC of the IT of P1 in ring 3 combined with that in ring 5 was 0.887.

## 4. Discussion

Diabetic retinopathy is one of the leading causes of blindness in developed countries. Pathophysiological studies have demonstrated that diabetic retinopathy is a disease of the small retinal vessels that develops even before the appearance of visible fundus lesions. Typical early changes in the retinal vasculature of diabetic eyes are pericyte loss, basement membrane thickening, microaneurysms, and hyperglycemia [14–16]. Local glucose levels regulate some factors associated with diabetic retinopathy that modulate the activity of smooth muscle cells and pericytes. Hyperglycemia does not lead to visible pathological changes in the retina within the first six weeks. However, even blood glucose levels return to normal, and pericyte apoptosis, basement membrane swelling, vascular endothelial cell proliferation, retinal neurons damage, and vessel lesions are observed. These processes are collectively called “metabiotic memory.” Thus, the identification of retinal abnormalities during the early disease stages of diabetic patients is critically important.

There is considerable evidence for defects in the retinal function in diabetic patients even at very early stage of diabetic retinopathy, before the appearance of pathological changes [17–19]. A characteristic example is an observed decline in the visual function, including color vision and contrast sensitivity, in diabetic patients, including those without the clinical manifestation of retinopathy. Microstructurally, the function of the retinal vasculature is also slowly damaged during the diabetes. Previous studies have reported an increase in retinal blood flow and heterogeneity in the distribution of retinal blood flow [20–22]. So, the diagnosis of DR is not simple, especially when it is in the early stage. Recently, Kim et al. [23] concluded that the retinal neurodegeneration and microvascular change may have high association in the early stage of DM. They identified the macular ganglion cell/inner plexiform layer (mGCIPL) thinning prior to the microvascular impairment in DR by optical coherence tomography angiography. That is the morphological evidence. And our research has illustrated this conclusion in the retinal function in DM patients.

Previous research has demonstrated changes in the implicit time and/or amplitude response in diabetic patients with or without retinopathy. Some studies have reported a decrease in mf-ERG amplitudes in diabetic patients [24, 25]. However, other groups have also described higher amplitudes of the first- and second-order components in diabetic patients, a phenomenon that is believed to depend on the higher retinal blood flow resulting from vascular abnormalities in diabetic patients [26, 27]. In the present study, we found that the amplitude response of the P1 wave tended to decrease in all rings; however, these changes had not a statistically significant difference (*p* > 0.05). This observation is consistent with previous research [28]. One possible explanation for our findings is that the recruited patients in this research have a steady, normal blood sugar level for a long duration, and their retinal functions were similar to that of a healthy person. Furthermore, in the early stage of diabetes without visible retinopathy, the mf-ERG results showed only selective neurosensory deficits in the inner layers of the retina. Another consideration is that measurement of the amplitude response reflects the strength of the summed responses generated by retinal cells, and changes in the amplitude response failed to reflect abnormalities in the retinal function within only a certain ring zone.

We found that the implicit time of the P1 and N1 waves was significantly prolonged in some regions of the retina in type 2 diabetic patients. The delayed implicit time in diabetic patients without clinically apparent retinopathy may be a consequence of early or undetected perfusion or retinal hypoxia defects associated with choriocapillary degeneration [29].

Additionally, in the present study, we also found that the function of temporal retina was more frequently affected than that in the nasal retina. It suggests that before the appearance of retinopathy, the function of the retina is more susceptible to be damaged. We also found that the greater susceptibility of the temporal retina may be attributed to the reduced retinal vasodilator reserve and the potentially associated risk of ischemic damage in comparison to the nasal retina [30–33]. And another possible reason is, as Curcio et al. reported, a higher density of cones and ganglion cells in the nasal macular area compared with the temporal area [32]. The mf-ERG recordings reflect the electrical activity of bipolar and photoreceptor cells. Our findings suggest that high blood sugar results in greater damage to the temporal retina in diabetic patients, even those without clinically apparent retinopathy, because the low density of cones and ganglion cells in the temporal area is associated with less compensatory function.

Interestingly, the mf-ERG amplitude measurements were not significantly decreased in rings compared with the implicit times, which is consistent with previous findings [28]. One possible explanation for this result is that focal ERG revealed selective neurosensory deficits of the inner retinal layers in patients with early-stage diabetes without visible retinopathy. So, these results are suggested that only one parameter in certain ring could not reflect the retinal dysfunction especially in the early stage of DM, and it should be combined with multiparameters to detect the abnormal change of retina. This is the new perspectives of our research.

The ROC analysis illustrated that although the IT in ring 1,ring 3, and ring 5 of the P1 wave had significant difference in DM patients, compared with the health control group, and the single parameter ROC curve analysis showed the low AUC, respectively.

Multiparameter ROC curve analysis demonstrated that it can increase the diagnostic efficacy of diabetic retinopathy. The combination of multiparameter mf-ERG could get a higher diagnosis effectiveness to identify diabetic retinopathy in DM patients without clinically visible DR in the early stage. It indicated that the IT in P1 combined with IT in P3 had the highest efficacy in diagnosis of DR. It reveals that the abnormal results of mf-ERG test in multiregions of the posterior retina may suggest the prominently decreased retinal function of DM patients even without apparent clinical diabetic retinopathy.

In summary, before the onset of clinically apparent diabetic retinopathy, there is a prolonged period of pathological changes. The mf-ERG is well suited to study retinal diseases that are confined to local lesions, especially those in the posterior pole such as diabetic retinopathy and age-related macular degeneration, among others. The mechanism underlying the mf-ERG implicit time delay in diabetic patients remains unknown. Hypoxia, modifications of local blood flow, or changes in local metabolism may be responsible for the observed effects. The mf-ERG implicit time is sufficiently sensitive to reflect the retinal function and can be employed to evaluate diabetic patients without apparent clinical retinopathy or those in very early stages of diabetes.

## Figures and Tables

**Table 1:** Epidemiologic and disease characteristics of all participants in this study.

Number of subjects	HC	DM	*p*
	*n* = 32	*n* = 38	
Age (years)	65.19 ± 5.46	64.08 ± 8.35	0.522^a^
Male/female	17/15	25/13	0.157^b^
DM duration (years)	—	7.13 ± 1.63	—
HbA1c (%)	4.9 ± 0.70	5.5 ± 0.80	0.005^a^^∗∗^
BCVA	4.97 ± 0.06	4.96 ± 0.71	0.245^c^
IOP (mmHg)	15.09 ± 2.57	14.29 ± 2.87	0.086^c^

Values expressed as mean ± SD (unless otherwise stated). Statistical tests: ^a^*t*-test, ^b^Pearson *х*^2^ test, and ^c^Mann–Whitney *U* test. ^∗∗^*p* < 0.01. BCVA: best corrected visual acuity; IOP: intraocular pressure.

**Table 2:** Retinal function analysis of DM patients and healthy subjects using mf-ERG examinations in healthy controls and DM patients.

Number of eyes tested	HC	DM	*p*
	*n* = 64	*n* = 76	
AD of P1 in ring 1	121.88 ± 16.46	116.70 ± 22.90	0.133
AD of P1 in ring 2	40.87 ± 13.95	37.59 ± 12.30	0.142
AD of P1 in ring 3	18.68 ± 3.31	18.56 ± 4.38	0.861
AD of P1 in ring 4	14.17 ± 4.70	12.96 ± 4.59	0.127
AD of P1 in ring 5	8.64 ± 2.00	7.94 ± 2.21	0.053
IT of P1 in ring 1	45.42 ± 4.74	48.85 ± 3.00	≤0.001^∗∗^
IT of P1 in ring 2	43.20 ± 5.26	44.22 ± 4.25	0.206
IT of P1 in ring 3	40.63 ± 3.53	44.81 ± 2.56	≤0.001^∗∗^
IT of P1 in ring 4	43.64 ± 3.29	44.75 ± 4.50	0.102
IT of P1 in ring 5	39.97 ± 4.04	41.05 ± 2.26	0.047^∗^
AD of N1 in ring 1	0.78 ± 0.26	0.78 ± 0.26	0.880
AD of N1 in ring 2	0.36 ± 0.16	0.34 ± 0.12	0.349
AD of N1 in ring 3	0.24 ± 0.07	0.23 ± 0.04	0.079
AD of N1 in ring 4	0.23 ± 0.05	0.24 ± 0.08	0.461
AD of N1 in ring 5	0.16 ± 0.12	0.16 ± 0.07	0.754
IT of N1 in ring 1	19.41 ± 4.85	18.59 ± 6.60	0.412
IT of N1 in ring 2	21.71 ± 3.79	23.25 ± 5.86	0.072
IT of N1 in ring 3	19.44 ± 5.28	19.40 ± 6.56	0.970
IT of N1 in ring 4	21.82 ± 7.07	23.92 ± 3.20	0.021^∗^
IT of N1 in ring 5	25.84 ± 4.69	24.12 ± 6.13	0.069

Values expressed as mean ± SD. Statistical tests: Mann–Whitney *U* test. AD: amplitude density (nV/deg^2^); IT: implicit time (ms). ^∗^*p* < 0.05, ^∗∗^*p* < 0.01.

## Data Availability

The data used to support the findings of this study are available from the corresponding author upon request.
